# Editorial: Multifunctional Bioactive Nanomaterials for Tissue Regeneration

**DOI:** 10.3389/fchem.2019.00679

**Published:** 2019-10-15

**Authors:** Bo Lei, Aldo R. Boccaccini, Xiaofeng Chen

**Affiliations:** ^1^Frontier Institute of Science and Technology, Xi'an Jiaotong University, Xi'an, China; ^2^Key Laboratory of Shaanxi Province for Craniofacial Precision Medicine Research, College of Stomatology, Xi'an Jiaotong University, Xi'an, China; ^3^State Key Laboratory for Manufacturing Systems Engineering, Xi'an Jiaotong University, Xi'an, China; ^4^Department of Materials Science and Engineering, Institute of Biomaterials, University of Erlangen-Nuremberg, Erlangen, Germany; ^5^Department of Biomedical Engineering, School of Materials Science and Engineering, South China University of Technology, Guangzhou, China; ^6^National Engineering Research Center for Tissue Restoration and Reconstruction, Guangzhou, China; ^7^Key Laboratory of Biomedical Materials and Engineering, South China University of Technology, Ministry of Education, Guangzhou, China

**Keywords:** bioactive nanomaterials, multifunctional biomaterials, tissue engineering, tissue regeneration, biomedical applications

In the biomedical field, human organ repair and regeneration represent a very important and challenging tasks (Christman, 2019). Many promising tissue regenerative strategies involve biomaterials in the form of engineered scaffolds. Conventional biomaterials are usually effective in enhancing tissue regeneration through the addition of cells and growth factors. However, recent studies have shown that biomaterials themselves can induce or support tissue regeneration without needing cells or the addition of growth factors, and these biomaterials are considered “bioactive.” Well-investigated and emerging bioactive biomaterials include bioactive glasses, bioactive ceramics, natural polymers such as proteins and polysaccharides, as well as a series of natural and synthetic biomaterials (and their combinations) with biological functions (anti-bacterial, anti-oxidant, anti-inflammatory, and anti-tumor etc.) (Lei et al., 2019; Li et al., 2019; Ranganathan et al., 2019; Schuhladen et al., 2019; Wang C. et al., 2019; Wang M. et al., 2019; Zhou et al., 2019). Relative to conventional biomaterials, nanoscale bioactive biomaterials applied in regenerative medicine have attracted increasing interest in recent years, due to their special nanoscale biological effects and enhanced capacity for tissue regeneration (Guo et al., 2018; Zhao et al., 2018; Niu et al., 2019; Wu et al., 2019; Xue et al., 2019).

Among bioactive nanomaterials, bioactive glass nanoparticles have shown special advantages including controlled biodegradation, controlled bioactive ion release, controlled multifunctional properties and high bioactivity, which render them highly attractive for both bone and soft tissue regeneration (Zheng and Boccaccini, 2017). Monodispersed bioactive glass nanoparticles with porous structure, for example, have been developed to load drugs and genes for cancer therapy and for enhancing bone formation (Xue et al., 2017; Yu et al., 2017). Through trace elements doping, it is possible to enable the tunable physicochemical and biological properties of bioactive glass nanoparticles such as photoluminescence (Xue et al., 2015), anti-bacterial (Vale et al., 2018), and anti-inflammatory capacity (Kim et al., 2019). Other bioactive nanomaterials including calcium phosphate and polymer nanoparticles have been reviewed by Loo et al. (2010) and (Torres-Giner et al., 2016).

Although the synthesis and biomedical applications of multifunctional bioactive nanomaterials have received much progress in recent years, challenges still exist, for example: (1) control of intrinsical multifunctional properties for enhanced bioactivity and drug/gene delivery; (2) Control of bioactivity and biodegradation; and (3) understanding molecular mechanism of nanomaterials controlling tissue regeneration. This Research Topic has attracted a series of papers that show the recent advances in the synthesis and biomedical application of bioactive nanomaterials especially bioactive glass nanoparticles, and provide new insights on designing bioactive nanomaterials. In this Research Topic, Zheng et al. reported the synthesis of dispersed mesoporous bioactive glass nanoparticles with high Cu concentration by an ascorbic acid complex precursor. Zhuang et al. reviewed the advance of nanocomposite electrospun fibers in periodontal regeneration. The advances of electrospraying for designing nanomaterials were also reviewed by Wang et al. In addition, Tian et al. and Wang et al. showed the preparation and properties of hollow mesoporous bioglass nanoparticles and micro-nano bioactive composites scaffolds for bone regeneration application. Lim et al. presented a novel multifunctional nanowire platform for highly isolation and analysis of circulating tumor specific markers.

The editors hope that the Research Topic “Multifunctional Bioactive Nanomaterials for Tissue Regeneration” will contribute to the progress of research and development activities in the field of novel bioactive nanomaterials for regenerative medicine, inspiring future work leading to the expansion of the biomedical applications of such bioactive nanomaterials.

## Author Contributions

BL proposed the Research Topic and editorial and in charge of 2 manuscript for review process. AB revised the topic and editorial. XC was in charge of 1 manuscript for review process. All authors listed have made a substantial, direct and intellectual contribution to the work.

### Conflict of Interest

The authors declare that the research was conducted in the absence of any commercial or financial relationships that could be construed as a potential conflict of interest.
